# A Wearable Haptic Feedback System for Arm-Swing Amplitude Modulation During Overground Walking in Older Adults

**DOI:** 10.3390/s26051532

**Published:** 2026-02-28

**Authors:** Ines Khiyara, Ben Sidaway, Babak Hejrati

**Affiliations:** 1 Biorobotics and Biomechanics Laboratory, Department of Mechanical Engineering, University of Maine, Orono, ME 04469, USA; babak.hejrati@maine.edu; 2 School of Physical Therapy, Husson University, Bangor, ME 04401, USA; sidawayb@husson.edu

**Keywords:** gait analysis, arm-swing range of motion, arm-swing symmetry, walking speed, stride length, vibrotactile feedback, inertial measurement units, closed-loop feedback, smartphone-based system

## Abstract

Reduced arm swing frequently occurs in older adults and is associated with declined gait performance. Experimental studies have demonstrated that restricting arm swing decreases stride length and walking speed, whereas deliberately increasing arm swing can improve these gait parameters. This study evaluated whether a wearable haptic feedback system could effectively increase arm-swing amplitude and assess its effects on spatiotemporal gait outcomes during overground walking. Using a within-subject repeated-measures design, twelve community-dwelling older adults (6 males/6 females; 75.8±6.5 years) completed three no-feedback conditions (Baseline, Exaggerated, Fast) and six feedback conditions varying *Direction* (Forward, Backward, Combined) and target *Magnitude* (+100%, +200% of the Baseline). The arm-swing angle was estimated in real time from upper-arm inertial measurement unit (IMU) sensors; targets were defined for peak Forward flexion and/or peak Backward extension, and vibrotactile cues were delivered when the corresponding peak failed to reach the target. The arm range of motion (ROM) increased significantly across conditions, with the largest increase during Feedback (+229%), exceeding Exaggerated (+120%) and Fast (+64%) (all p<0.001). Walking speed and stride length also increased during Feedback relative to the Baseline (p<0.001). Within feedback conditions, the arm ROM showed independent main effects of the *Direction* and *Magnitude*, whereas gait outcomes were primarily influenced by *Direction*. Arm-swing symmetry was largely preserved, with the smallest variability during Feedback. These findings support the feasibility of vibrotactile feedback to enhance arm swing and improve gait outcomes in older adults.

## 1. Introduction

Wearable sensing systems are increasingly used for ambulatory gait monitoring and rehabilitation, enabling quantitative assessment and real-time intervention outside of laboratory environments [1,2]. Inertial measurement unit (IMU) sensors, in particular, offer a lightweight and scalable solution for capturing human motion during overground walking [3], making them well suited for continuous real-time monitoring and feedback applications [4,5,6]. While IMU-based gait analysis has advanced considerably, real-time closed-loop systems that actively guide user movement remain limited [1,2]. Implementing real-time closed-loop control in ambulatory environments demands robust sensing as well as computationally efficient, low-latency processing executed locally on portable hardware [7,8,9]. Recent work has demonstrated that interpretable biomechanics-derived features extracted from a small number of IMU sensors can support accurate real-time classification on smartphones, enabling low-latency deployment in older-adult populations [7]. Such evidence supports the feasibility of mobile, resource-efficient wearable systems as practical platforms for real-time gait monitoring and intervention [1,4,7].

Haptic feedback is a promising modality for real-time gait intervention because it does not rely on visual or auditory attentional processing and can be delivered unobtrusively during typical walking [9,10,11]. Prior wearable haptic systems have primarily focused on temporal cueing (e.g., step timing) or binary event-based feedback [10,12]. However, these approaches fail to directly regulate movement amplitude and typically depend on fixed events or generalized instructions [10], limiting their scalability across individuals and task conditions [1,2]. In contrast, parameter-focused haptic feedback that targets specific biomechanical variables may provide a more individualized training strategy [6,13]. For example, recent work applying real-time vibrotactile feedback to increase peak thigh extension demonstrated significant improvements in stride length and gait speed in older adults, while imposing minimal additional cognitive load [13]. Beyond spatiotemporal outcomes, haptic feedback has also been shown to induce coordination-level adaptations; for example, thigh-extension haptic feedback altered lower-limb intersegmental coordination and increased coordination variability in both young and older adults, suggesting neuromuscular reorganization in response to tactile error feedback cues [14]. These findings demonstrate the feasibility and acceptability of wearable haptic feedback as a means of modulating gait-related biomechanics in community-dwelling older adults [6,13].

Efficient locomotion emerges from coordination dynamics that organize neuromotor coupling and mechanically link arm and leg movements [15,16,17,18]. Older adults commonly exhibit reduced or asymmetric arm swing, which has been associated with slower gait and impaired stability [15,19]. Experimental manipulation of arm-swing amplitude has been shown to directly influence gait, with arm-swing restriction reducing stride length and walking speed and arm-swing enhancement producing the opposite effect [20,21,22,23]. These findings are clinically important because age-related reductions in walking speed are strong predictors of mobility limitation, fall risk, and health outcomes in older adults [24,25,26,27]. Therefore, interventions that can safely increase stride length and consequently speed during normal walking have high translational relevance [13,28], especially by increasing arm swing or improving symmetry. Previous studies have shown that voluntary exaggeration of arm swing and rhythm-based arm-swing training can influence spatiotemporal gait parameters [12,29], such as increasing speed, step length and variability [30]. Separately, increased arm-swing amplitude has also been linked to improvements in gait stability, including mediolateral trunk stability [30,31]. However, these voluntary approaches may increase attentional demands, which could limit their feasibility for sustained use during everyday walking, particularly in older adults who exhibit pronounced dual-task effects on arm-swing amplitude and symmetry [19]. Wearable technologies have emerged as promising approaches for arm-swing training. Recent developments have explored wearable assistive devices designed to facilitate arm swing through physical guidance, underscoring the growing interest in integrating arm swing into gait rehabilitation. For instance, a prototype arm-swing facilitator device was shown to generate sufficient shoulder torque to induce cyclic arm motion in healthy young adults at gait-relevant frequencies, establishing feasibility for future rehabilitation-oriented designs [32]. Moreover, wearable haptic systems have been used to regulate arm swing during walking. For example, Lee et al. used wearable vibrotactile bracelets to regulate arm swing in young adults during *overground* walking, demonstrating that increasing arm swing can increase stride length and influence gait symmetry with direction-specific effects [23]. More recently, Fan et al. proposed an IMU-based real-time biofeedback wristband capable of estimating arm-swing kinematics online and delivering vibrotactile cues when target amplitude is not achieved in young adults, with validation performed during *treadmill* walking [33]. Collectively, these efforts suggest that arm swing is an actionable and clinically relevant target for wearable intervention; however, real-time systems capable of quantitatively regulating arm-swing amplitude targets during overground walking, particularly in older adults, are still lacking.

To address this gap, we developed a wearable, IMU-driven haptic (vibrotactile) feedback system that monitors arm-swing amplitude in real time and delivers error-based vibration cues when movement fails to reach predefined individualized targets. Unlike timing-based or instruction-only approaches, the proposed system provides continuous, amplitude-specific feedback, allowing users to self-correct movement based on quantitative sensor measurements. The system supports flexible configuration of feedback *Direction* (flexion, extension, or Combined) and target *Magnitude*, aligning with ongoing efforts to individualize wearable gait interventions [1,23].

The purpose of this feasibility study was to evaluate whether a fully portable, smartphone-based closed-loop vibrotactile feedback system could increase arm-swing amplitude during overground walking in older adults and examine the resulting short-term changes in spatiotemporal gait measures, as an initial exploratory investigation of system responsiveness rather than clinical effectiveness. Specifically, arm-swing kinematics were estimated in real time from wearable IMU sensors and used to deliver direction-specific vibrotactile cues when arm-swing amplitude failed to reach individualized amplitude targets (+100% and +200% of the Baseline). We hypothesized that closed-loop feedback would produce larger increases in the arm-swing range of motion (ROM) than two verbal instruction conditions (Exaggerated arm swing and Fast walking), and that these changes would be accompanied by systematic alterations in spatiotemporal gait measures (walking speed and stride length). As a secondary aim, we examined how feedback parameters influenced outcomes by testing whether feedback *Direction* (Forward, Backward, Combined) and target *Magnitude* (+100%, +200%) produced distinct and target-dependent changes in arm kinematics and gait outcomes. We further hypothesized that the *Direction* and *Magnitude* of feedback would independently influence arm-swing kinematics, with Combined feedback yielding the largest increases in total arm ROM and +200% eliciting greater kinematic and gait changes than +100%.

## 2. Materials and Methods

### 2.1. Study Design

The experiment was designed to evaluate whether real-time vibrotactile feedback with varying directions and target magnitudes could systematically increase arm-swing amplitude and influence gait outcomes relative to no-feedback walking conditions. This feasibility study employed a within-subject repeated-measures experimental design in which all participants completed nine walking conditions, including three no-feedback conditions and six real-time feedback conditions:

- No-feedback conditions: Baseline, Exaggerated, and Fast.
- Feedback conditions: Forward +100%, Forward +200%, Backward +100%, Backward +200%, Combined +100%, and Combined +200%.

The feedback conditions systematically varied two within-subject factors—feedback *Direction* (Forward swing/flexion, Backward swing/extension, or Combined/bidirectional) and target *Magnitude* (2× [+100%] or 3× [+200%] relative to the Baseline arm-swing amplitude).

Direction-specific feedback was implemented to target distinct phases of the arm-swing cycle. Forward-only feedback targeted peak flexion, Backward-only feedback targeted peak extension, and Combined feedback targeted both phases. This design enabled the systematic evaluation of whether specific swing phases differentially influence gait outcomes, whether bidirectional feedback produces different effects than single-direction feedback, and whether feedback *Direction* and *Magnitude* contribute independently or interactively to arm-swing modulation and gait performance. Forward flexion and Backward extension are governed by distinct neuromuscular mechanisms and contribute differently to interlimb coordination and gait mechanics [18,34,35], and prior work has demonstrated distinct effects of directional arm-swing cues on gait performance [23]. Target magnitudes of 2× (+100%) and 3× (+200%) Baseline were selected as participant-specific proportional increases rather than fixed absolute angles. This approach is consistent with prior vibrotactile arm-swing feedback work in young adults, in which Lee et al. [23] instructed participants to double their Baseline arm-swing amplitude and observed significant increases in stride length. Building on this precedent, the +100% target was included as a literature-supported modulation level, while the +200% target was added to examine whether larger proportional increases would produce greater arm-swing modulation and gait changes in older adults.

### 2.2. Participants

Inclusion criteria were age ≥65 years and the ability to walk independently for at least 20 minutes. Exclusion criteria included neurological disorders, cognitive impairment, or musculoskeletal conditions affecting gait. Twelve community-dwelling older adults (aged ≥65 years) who met these criteria participated in this study (mean age: 75.8±6.5 years; 6 males/6 females). Participants had a mean height of 170.1±12.7 cm, body mass of 66.7±14.7 kg, and body mass index of 22.8±2.3 kg/m^2^. Ten participants reported right-side dominance, and two reported left-side dominance. Functional mobility was assessed using the Short Physical Performance Battery (SPPB) [36], with a mean score of 10.6±1.2 out of 12, indicating high physical function. Balance confidence was evaluated using the Activities-specific Balance Confidence (ABC) scale [37], with a mean score of 86.9%. All participants provided written informed consent prior to participation, and any personal information and data collected were anonymized to ensure confidentiality. The experimental protocol was approved by the University of Maine Institutional Review Board.

### 2.3. System Overview

**Figure 1:**
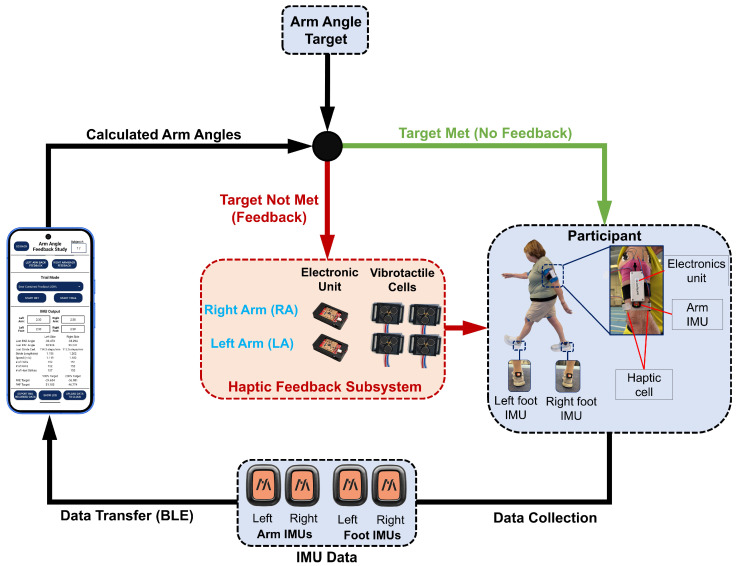
Closed-loop vibrotactile feedback system for real-time arm-swing modulation during overground walking. Four wireless IMU sensors (two arm-mounted, two foot-mounted) stream data via Bluetooth Low Energy (BLE) to a smartphone application, which computes arm-swing angles in real time. Calculated arm angles are compared with individualized amplitude targets. When the target is met, no feedback is delivered (green arrows). When the target is not met, the smartphone triggers the haptic feedback subsystem (red arrows) to deliver vibrotactile stimulation to the corresponding arm via bilateral electronic units and vibrotactile cells. The participant view shows the placement of IMU sensors, electronic units, and vibrotactile cells during walking.

A portable closed-loop system was developed to provide real-time vibrotactile feedback for arm-swing modulation during overground walking (Figure 1). During each trial, participants walked overground while wearing IMU sensors on the upper arms and feet, as well as bilateral vibrotactile feedback modules. The system continuously estimated arm angles and delivered vibrotactile cues when arm-swing parameters did not meet the condition-specific target. A Google Pixel 10 smartphone (Google LLC, Mountain View, CA, USA) served as the central controller and processing unit. A custom Android application was developed to (i) stream IMU signals via Bluetooth Low Energy (BLE), (ii) compute real-time kinematic outcomes (e.g., arm-swing angle and gait events), and (iii) control feedback delivery. Closed-loop control was implemented by comparing the estimated arm-swing angle against an individualized condition-specific target. When the target was not met, the controller triggered vibrotactile stimulation on the corresponding arm (right or left) through wearable electronic units connected to vibrotactile cells. When the target was met, vibration was not delivered. Feedback decisions (on/off and side selection) were updated continuously throughout walking to provide immediate sensory cues guiding arm-swing modulation.

### 2.4. Wearable System Components and Placement

Upper- and lower-limb kinematics were measured using wireless IMU sensors (Movella DOT, Movella Inc., Henderson, NV, USA). The Movella DOT IMUs provide orientation estimates with a reported dynamic orientation error of approximately 1° RMS, as specified by the manufacturer [38]. Independent validation studies have also demonstrated strong agreement between Movella/Xsens IMU systems and optical motion capture for upper-limb segment tracking (RMSE $\approx 1–3^{\circ }$) during functional movements [39,40,41]. Four IMU sensors were used for real-time processing: two mounted bilaterally on the upper arms (mid-brachium) to quantify arm swing, and two mounted on the feet to support gait event detection and capture gait kinematics. Arm-mounted IMU sensors streamed complete orientation as quaternions (measurement mode 3), which were converted on-device to Euler angles using a ZYZ rotation sequence to extract the sagittal-plane arm-swing angle. Foot-mounted IMU sensors streamed Euler angles directly (custom mode 1, ZYX sequence), from which sagittal-plane pitch was extracted for heel-strike detection to define gait cycles and enable estimation of spatiotemporal gait parameters, including cadence, stride length, and walking speed. All sensors streamed at 60 Hz.

Vibrotactile feedback was delivered using bilateral wearable haptic modules mounted on the upper arms using Velcro straps, consistent with our prior wearable cueing setup [12]. Each module consisted of a compact electronic unit housed in a 3D-printed enclosure and included an ESP8266 microcontroller (Espressif Systems, Shanghai, China), a rechargeable battery, and a custom circuit board for driving the vibrotactile cells. The Pixel smartphone transmitted control commands to the electronic units as HTTP requests over Wi-Fi, with the ESP8266 microcontrollers functioning as HTTP servers connected to a Wi-Fi network initiated by the smartphone [12,13]. These commands specified which vibration cell(s) to activate/deactivate in real time. Each vibrotactile cell incorporated three vibrotactors that vibrated at approximately 240 Hz, a frequency near the resonant frequency of Pacinian mechanoreceptors, producing a comfortable and distinctly perceivable tactile sensation [12,13,42]. As seen in Figure 1, each arm included two vibrotactile cells to provide direction-specific stimulation: one positioned on the anterior aspect of the brachium to cue Forward swing (flexion) and one positioned on the posterior aspect of the brachium to cue Backward swing (extension). This dual-cell configuration enabled Forward, Backward, and Combined feedback conditions by activating the corresponding cell(s) when the arm-swing angle deviated from the target.

### 2.5. Sensor Initialization and Reference Frame Definition

At the beginning of the experimental session, an initialization procedure was performed following a synchronized heading reset across all sensors. Participants stood upright in a neutral posture with their arms relaxed at their sides for 3 s (180 samples at 60 Hz), during which orientation offsets were computed (Algorithm 1). These offsets were computed once per session and applied consistently across all subsequent trials.
**Algorithm 1** Session initialization and offset computation
1: Let $f_{s}=60$ Hz and N=180
2: **for** i=1 to *N* **do**
3:    Acquire orientation data
4:    Compute         $$\theta_{i}=\left\{\begin{array}{c} \theta_{\mathrm{arm}}\left(q_{i}\right),\;\;\;\text{arm-mounted}\;\mathrm{sensors}\\ \theta_{\mathrm{foot}}\left(q_{i}\right),\;\;\;\text{foot-mounted}\;\mathrm{sensors} \end{array}\right.$$
5: **end for**
6: Compute session offset:               $\theta_{\mathrm{offset}}=\frac{1}{N}\sum_{i=1}^{N}\theta_{i}$
7: Apply $\theta_{\mathrm{offset}}$ to all subsequent samples


### 2.6. Real-Time Arm-Swing Angle Estimation

Arm-swing angles were estimated as the sagittal-plane orientation of the upper-arm segment (brachium) with respect to the global (Earth) reference frame using Movella DOT IMU sensors, which provide real-time orientation data through commercially implemented sensor fusion algorithms. Prior to testing, a functional verification step was performed in which participants raised the arm to approximately 90° of shoulder flexion and actively extended the arm Backward to confirm that the measured angles corresponded to the expected positive and negative anatomical directions. Arm-swing angles were computed in real time from quaternion orientation data using a ZYZ rotation sequence selected to represent sagittal-plane rotation for laterally mounted arm sensors (Algorithm 2).
**Algorithm 2** Real-time arm-swing angle estimation
  1: Acquire quaternion $q=[q_{0},q_{1},q_{2},q_{3}]$
  2: **if** 
∥q∥<0.001 
**then**
  3:    Discard sample
  4: **end if**
  5: Convert quaternion to rotation matrix $R=[r_{ij}]$
  6: Compute:$$\alpha =\arctan2(r_{23},r_{13})$$$$\gamma =\arctan2(r_{21}\cos\alpha -r_{11}\sin\alpha ,\;r_{22}\cos\alpha -r_{12}\sin\alpha )$$
  7: Convert to degrees: $\gamma \leftarrow \gamma \cdot \frac{180}{\pi }$
  8: Normalize $\gamma \in [-180^{\circ },180^{\circ }]$
  9: Offset correction: $\gamma \leftarrow \gamma -\theta_{\mathrm{offset}}$
10: Enforce symmetry: γ←−γ for left arm


### 2.7. Arm-Swing Peak Detection

Peak flexion (Forward swing) and peak extension (Backward swing) events were identified using a five-sample sliding-window approach applied to the offset-corrected arm-swing angle (Algorithm 3). A minimum separation criterion was enforced to prevent false detections. Due to window centering, the peak detection window was centered at index k−2 relative to the current sample *k*. For arm-mounted sensors used in real-time feedback control, peaks were recorded at the current sample index *k* to minimize feedback latency.
**Algorithm 3** Arm-swing peak detection
  1: Let γ(k) be the offset-corrected arm-swing angle at sample *k*
  2: Window size W=5 samples, minimum separation S=30 samples
  3: Thresholds: $\tau_{\mathrm{flex}}=3^{\circ }$, $\tau_{\mathrm{ext}}=-2^{\circ }$
  4: Initialize $k_{\mathrm{last},\mathrm{flex}}\leftarrow 0$ and $k_{\mathrm{last},\mathrm{ext}}\leftarrow 0$
  5: **for** each new sample k≥5 **do**
  6:    Form window γ(k−4:k) and set candidate index $k^{★}=k-2$
  7:    **if** k>180
**and**
$\gamma \left(k^{★}\right)=\max\left(\gamma \left(k-4:k\right)\right)\wedge \gamma \left(k^{★}\right)>\tau_{\mathrm{flex}}\wedge \left(k^{★}-k_{\mathrm{last},\mathrm{flex}}\right)\geq S$ **then**
  8:      Detect flexion peak at $k^{★}$
  9:      $k_{\mathrm{last},\mathrm{flex}}\leftarrow k^{★}$
10:    **end if**
11:    **if** k>180
**and**
$\gamma \left(k^{★}\right)=\min\left(\gamma \left(k-4:k\right)\right)\wedge \gamma \left(k^{★}\right)<\tau_{\mathrm{ext}}\wedge \left(k^{★}-k_{\mathrm{last},\mathrm{ext}}\right)\geq S$ **then**
12:      Detect extension peak at $k^{★}$
13:      $k_{\mathrm{last},\mathrm{ext}}\leftarrow k^{★}$
14:    **end if**
15:**end for**
**Implementation note:** With a centered window, a peak at $k^{★}=k-2$ becomes detectable when sample *k* is acquired (2-sample delay). For foot-mounted sensors, events were time-stamped at $k^{★}$ for gait event timing. For arm-mounted sensors, vibrotactile feedback was triggered at the detection instant (*k*) to minimize latency.  

The closed-loop feedback system requires individualized Baseline arm-swing amplitudes to define participant-specific targets. These Baseline values were established from a comfortable-pace walking trial performed before any feedback intervention, during which participants walked naturally without instruction. Baseline arm-swing amplitudes were computed from the peak flexion and peak extension angles detected during this trial. The flexion Baseline was defined as the larger of the left and right mean peak flexion angles, while the extension Baseline was defined as the more negative of the left and right mean peak extension angles:

(1) $$\theta_{\mathrm{baseline},\mathrm{flex}}=\max\;\left({\bar{\theta }}_{L,\mathrm{flex}},\;{\bar{\theta }}_{R,\mathrm{flex}}\right),$$

(2) $$\theta_{\mathrm{baseline},\mathrm{ext}}=\min\;\left({\bar{\theta }}_{L,\mathrm{ext}},\;{\bar{\theta }}_{R,\mathrm{ext}}\right).$$

These Baseline values were then used to compute condition-specific target angles for subsequent feedback conditions. The full sequence and characteristics of all walking conditions used in this study are described in Section 2.10.

### 2.8. Real-Time Vibrotactile Feedback Control

Real-time vibrotactile feedback was generated based solely on detected arm-swing peaks and was independent of gait event detection and spatiotemporal gait measures (Algorithm 4). To avoid transient effects at trial onset, feedback delivery was inhibited for the first 10 detected peaks of each type (extension and flexion counted separately) and enabled starting from the 11th peak.
**Algorithm 4** Real-time error feedback control with tolerance band and steady-state gating
  1: Initialize $c_{\mathrm{ext}}\leftarrow 0$, $c_{\mathrm{flex}}\leftarrow 0$
  2: Let *k* denote the sample index at which a peak occurred
  3: Let $\varepsilon =3^{\circ }$ denote the tolerance band
  4: Warm-up: inhibit extension feedback until $c_{\mathrm{ext}}>10$; inhibit flexion feedback until $c_{\mathrm{flex}}>10$
  5: **for** each detected extension peak at sample index *k* **do**
  6:    $c_{\mathrm{ext}}\leftarrow c_{\mathrm{ext}}+1$
  7:    **if** $c_{\mathrm{ext}}\leq 10$ **then**
  8:      continue {warm-up period}
  9:    **end if**
10:    Measure peak extension angle $\gamma_{\mathrm{ext}}$
11:    **if** condition includes Backward feedback (Backward or Combined) **then**
12:      **if** $\gamma_{\mathrm{ext}}>\gamma_{\mathrm{target},\mathrm{ext}}+\varepsilon$ **then**
13:         Trigger 500 ms Backward vibration
14:         Encode event: $\mathrm{ID}=10^{8}+\left(c_{\mathrm{ext}}\times 10^{5}\right)+k$
15:      **end if**
16:    **end if**
17: **end for**
18: **for** each detected flexion peak at sample index *k* **do**
19:    $c_{\mathrm{flex}}\leftarrow c_{\mathrm{flex}}+1$
20:    **if** $c_{\mathrm{flex}}\leq 10$ **then**
21:      continue {warm-up period}
22:    **end if**
23:    Measure peak flexion angle $\gamma_{\mathrm{flex}}$
24:    **if** condition includes Forward feedback (Forward or Combined) **then**
25:      **if** $\gamma_{\mathrm{flex}}<\gamma_{\mathrm{target},\mathrm{flex}}-\varepsilon$ **then**
26:         Trigger 500 ms Forward vibration
27:         Encode event: $\mathrm{ID}=10^{8}+\left(c_{\mathrm{flex}}\times 10^{5}\right)+k$
28:      **end if**
29:    **end if**
30: **end for**


During feedback conditions, vibrotactile cues (500 ms pulses) were delivered when peak arm-swing amplitude fell short of the condition-specific target by more than a tolerance band ($\varepsilon =3^{\circ }$). Target peak angles ($\gamma_{\mathrm{target},\mathrm{flex}}$, $\gamma_{\mathrm{target},\mathrm{ext}}$) were defined for each condition based on Baseline comfortable-walking peak angles and the assigned modulation level (+100% or +200%). Condition-specific target peak angles were set as:

(3) $$\begin{array}{cc} +100\%\;\mathrm{condition}: & \theta_{\mathrm{target},\mathrm{flex}}=2\times \theta_{\mathrm{baseline},\mathrm{flex}}\;;\;\theta_{\mathrm{target},\mathrm{ext}}=2\times \theta_{\mathrm{baseline},\mathrm{ext}}\\ +200\%\;\mathrm{condition}: & \theta_{\mathrm{target},\mathrm{flex}}=3\times \theta_{\mathrm{baseline},\mathrm{flex}}\;;\;\theta_{\mathrm{target},\mathrm{ext}}=3\times \theta_{\mathrm{baseline},\mathrm{ext}} \end{array}$$

Direction-specific control logic was applied:

- **Backward (extension) conditions:** Backward vibration was triggered when peak extension was insufficiently negative, i.e., $\gamma_{\mathrm{ext}}>\gamma_{\mathrm{target},\mathrm{ext}}+\varepsilon$ (extension angles are negative).
- **Forward (flexion) conditions:** Forward vibration was triggered when peak flexion was insufficiently positive, i.e., $\gamma_{\mathrm{flex}}<\gamma_{\mathrm{target},\mathrm{flex}}-\varepsilon$.
- **Combined conditions:** Vibration was triggered in either direction when the corresponding flexion or extension criterion was not met.

Feedback was triggered only when the peak error exceeded the tolerance band; no feedback was delivered when peaks met or exceeded the target within tolerance.

### 2.9. Real-Time Gait Event Detection and Spatiotemporal Estimation

Heel strikes were identified in real time from the right foot-mounted IMU using the offset-corrected sagittal-plane foot pitch angle. A five-sample sliding-window peak detector was applied to the sagittal-plane foot pitch angle to identify local maxima exceeding 10°, with a minimum separation of 15 samples between consecutive events. Cadence and gait cycle time were computed from consecutive heel-strike timestamps. Stride length was estimated in real time using a zero-velocity update approach [3,6] applied to the foot IMU free acceleration signals implemented in the Android application. Foot-flat phases were identified as the sample within each stride where the sagittal-plane foot pitch angle was closest to 0° with minimal angular variation across a five-sample window. Free acceleration signals were integrated using the trapezoidal method to obtain velocity over segments spanning three consecutive foot-flat events. Integration drift was corrected by enforcing zero-velocity constraints at each foot-flat event. The drift-corrected velocity was integrated a second time to obtain horizontal displacement, from which stride length was computed as the two-dimensional displacement magnitude between consecutive foot-flat events of the same foot. Walking speed was calculated as the stride length divided by gait cycle time. These real-time spatiotemporal measures were used for in-trial monitoring and were not used as inputs to the haptic feedback controller, which operated exclusively on arm-swing peaks (Algorithm 3). All spatiotemporal outcomes reported in the Results section were recomputed offline in MATLAB R2025b (MathWorks, Natick, MA, USA) using steady-state walking cycles (see Section 2.11).

### 2.10. Experimental Protocol

After system setup and initialization, participants completed all walking trials overground on a 200 m indoor stadium track, performing one trial per condition. Three initial conditions were completed without haptic feedback to characterize natural and instruction-based gait behavior:

1. **Baseline**, in which participants were instructed to “walk normally at a comfortable pace.”
2. **Exaggerated**, in which participants were instructed to “walk with exaggerated arm swing” without additional guidance or physical cueing.
3. **Fast**, in which participants were instructed to “walk as fast as possible while remaining comfortable and safe.”

Each no-feedback trial lasted 2 min. Following these conditions, participants took an extended seated rest break to minimize fatigue and potential carryover effects prior to the feedback trials. During this break, participants completed demographic and health history forms and the ABC scale.

Six additional walking conditions were then performed with real-time haptic feedback:

1. **Forward +100%:** Vibrotactile feedback during the Forward (flexion) phase of arm-swing targeting 2× Baseline amplitude.
2. **Forward +200%:** Vibrotactile feedback during the Forward (flexion) phase of arm-swing targeting 3× Baseline amplitude.
3. **Backward +100%:** Vibrotactile feedback during the Backward (extension) phase of arm-swing targeting 2× Baseline amplitude.
4. **Backward +200%:** Vibrotactile feedback during the Backward (extension) phase of arm-swing targeting 3× Baseline amplitude.
5. **Combined +100%:** Vibrotactile feedback during both Forward and Backward phases of arm-swing targeting 2× Baseline amplitude.
6. **Combined +200%:** Vibrotactile feedback during both Forward and Backward phases of arm-swing targeting 3× Baseline amplitude.

Each feedback trial lasted 3 min. The order of the six feedback conditions was counterbalanced across participants to minimize order effects, while no-feedback trials were performed in a fixed order. Short standing rest breaks were provided between walking trials for the feedback conditions as needed. Participants were instructed to walk naturally and respond to the vibration feedback by increasing their arm swing slightly in that direction during the next swing so that vibration would not be received.

**Figure 2:**
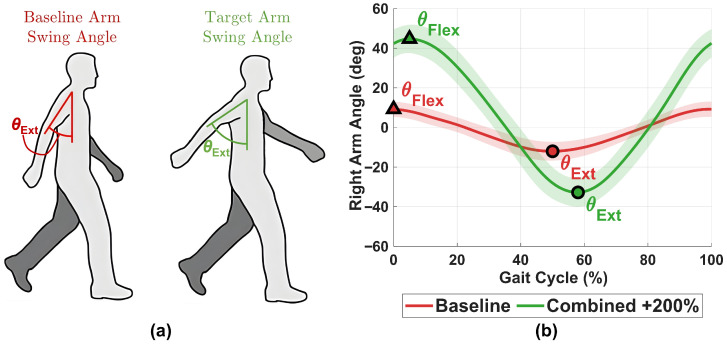
Arm-swing peak extraction and modulation during vibrotactile feedback. (**a**) Conceptual illustration of the Baseline peak extension angle ($\theta_{\mathrm{ext}}$) and the corresponding scaled target extension angle used for feedback control. (**b**) Group-averaged right-arm sagittal-plane trajectories during Baseline (red) and Combined +200% (green) walking over a normalized gait cycle (0–100%), illustrating changes in flexion and extension amplitudes under bidirectional (Combined +200%) feedback. Shaded bands represent 95% confidence intervals around the group mean.

Figure 2 illustrates the arm-swing modulation approach used during the Combined +200% feedback condition. Figure 2a conceptually depicts the Baseline peak extension angle and the corresponding target extension angle (denoted as $\theta_{\mathrm{ext}}$ for visualization). Figure 2b shows group-averaged right-arm sagittal-plane trajectories during Baseline and Combined +200% walking conditions, illustrating the modulation of arm-swing flexion and extension amplitudes under bidirectional feedback.

### 2.11. Data Processing

To ensure steady-state walking, the last 60 gait cycles of each trial were analyzed offline using MATLAB R2025b, excluding the final 5 cycles to avoid end-of-trial slowing. Heel strikes were identified from the sagittal-plane foot angle, and gait cycles were defined as consecutive heel-strike events. Gait cycle time was defined as the time interval between consecutive heel strikes. Each gait cycle was time-normalized to 0–100% to enable ensemble averaging across cycles and participants. Stride length was estimated from the foot-mounted IMU acceleration signal using an acceleration-integration approach with a zero-velocity update to reduce integration drift, consistent with prior IMU-based gait estimation methods [3,6]. Foot-flat phases were identified as the sample within each interval between the heel strike and the subsequent toe-off at which the sagittal-plane foot pitch angle was closest to $0^{\circ }$. Acceleration signals were integrated to obtain velocity, and drift was corrected by enforcing the zero-velocity constraint at consecutive foot-flat events using segment-wise correction. The corrected velocity was then integrated to obtain foot position, and stride length was computed as the two-dimensional horizontal displacement between consecutive foot-flat events of the same foot. Walking speed was calculated as stride length divided by the corresponding gait cycle time (heel-strike to heel-strike), and cadence (steps/min) was calculated from the gait cycle time. Stride length and walking speed were normalized to each participant’s height by dividing the measured values by individual height [6,12]. Height-normalized metrics were used for the primary statistical analyses, while absolute (non-normalized) stride length (m) and walking speed (m/s) are additionally reported. Arm-swing kinematics were computed from the sagittal-plane arm angle (Z-axis) for each arm sensor. Peak flexion and peak extension were identified within each gait cycle, with the arm ROM being computed by the difference between the maximum (peak flexion) and minimum (peak extension) angles. The right- and left-arm ROM were computed separately, and a Combined arm ROM was obtained as the average of the right and left values. Arm-swing symmetry was quantified as the right-to-left arm ROM ratio (R/L):

$${\mathrm{ROM}}_{\mathrm{ratio}}=\frac{{\mathrm{ROM}}_{\mathrm{Right}\;\mathrm{Arm}}}{{\mathrm{ROM}}_{\mathrm{Left}\;\mathrm{Arm}}}.$$

### 2.12. Statistical Analysis

Statistical analyses were performed in MATLAB R2025b using repeated-measures models and were cross-checked in IBM SPSS Statistics (Version 31; IBM Corp., Armonk, NY, USA). The dependent variables (outcomes) included the Combined arm ROM, arm-swing symmetry (ROM ratio R/L), normalized walking speed, normalized stride length, and cadence. For each outcome measure, values were computed for individual gait cycles and then averaged across the 60 cycles analyzed for each participant within each condition to obtain a single participant-level mean used in all statistical analyses. Normality of dependent variables was assessed using the Shapiro–Wilk test (α=0.05) for each outcome within each condition prior to parametric analyses. Sphericity was assessed using Mauchly’s test; when violated (p<0.05), Greenhouse–Geisser corrected degrees of freedom were used. Outcomes were first evaluated under four within-subject conditions: Baseline, Exaggerated, Fast, and Feedback. To construct the single feedback condition, each outcome measure was calculated separately for each of the six feedback conditions (Forward +100%, Forward +200%, Backward +100%, Backward +200%, Combined +100%, and Combined +200%), and then averaged within each participant to obtain a single participant-specific feedback value. A one-way repeated-measures ANOVA was conducted with *Condition* as the within-subject factor (4 levels). Significant main effects were followed by Bonferroni-adjusted pairwise comparisons. Effect sizes are reported as partial eta-squared ($\eta_{p}^{2}$). Statistical significance was set at α=0.05. To examine the independent contributions of feedback configuration parameters, outcomes within the six feedback conditions were additionally analyzed using factorial (two-way) repeated-measures ANOVA with feedback *Direction* (Forward, Backward, Combined) and feedback *Magnitude* (+100%, +200% of Baseline targets) as within-subject factors, including the *Direction* × *Magnitude* interaction. Significant main effects were followed by Bonferroni-adjusted post hoc pairwise comparisons. In addition to repeated-measures models, one-sample *t*-tests were used to assess whether the ROM ratio differed from perfect symmetry (R/L =1.0) within each no-feedback condition (Baseline, Exaggerated, Fast, Feedback) and within each feedback condition.

A sensitivity (post hoc) power analysis was conducted using G*Power (version 3.1.9.7) [43] for the repeated-measures ANOVA (within-subject factors). The analysis assumed α=0.05, power = 0.80, four repeated measurements, a correlation among repeated measures of 0.5, and a nonsphericity correction of 1. The results indicated that the sample size (n=12) was sufficient to detect a minimum effect size of f=0.36, corresponding to a medium-to-large effect ($\eta_{p}^{2}=0.113$).

## 3. Results

### 3.1. Effects of Walking Condition on Arm Swing and Gait

**Figure 3:**
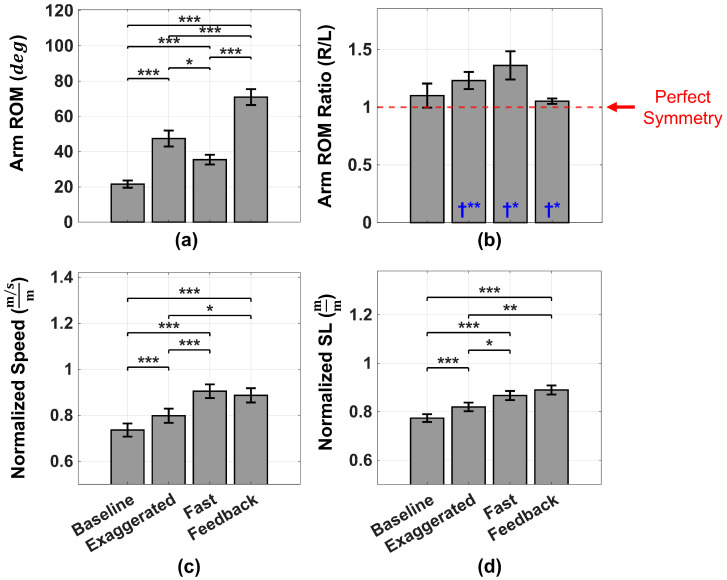
Effects of walking condition (Baseline, Exaggerated, Fast, and Feedback) on (**a**) arm-swing range of motion (ROM), (**b**) right-to-left arm ROM ratio (R/L), (**c**) normalized walking speed, and (**d**) normalized stride length (SL). Bars represent mean ± SEM. Horizontal brackets indicate significant Bonferroni-adjusted pairwise comparisons (* p<0.05, ** p<0.01, *** p<0.001). Dashed line in (**b**) indicates perfect symmetry (R/L = 1.0), and blue † symbols indicate significant deviation from 1.0 based on one-sample *t*-tests, with * and ** indicating p<0.05 and p<0.01, respectively.

All dependent variables met normality assumptions across all conditions (Shapiro–Wilk tests, all p≥0.05). Figure 3 summarizes the effects of conditions (Baseline, Exaggerated, Fast, and Feedback) on normalized gait metrics and arm-swing outcomes. Combined arm-swing ROM (averaged across right and left arms) differed significantly across conditions (repeated-measures ANOVA, F(3,33)=107.25, p<0.001, $\eta_{p}^{2}=0.907$). The ROM was greater during Exaggerated, Fast, and Feedback relative to the Baseline (all Bonferroni-adjusted p<0.001), and was highest during Feedback, which exceeded all other conditions (all p<0.001; Figure 3a). Relative to the Baseline, the Combined arm ROM increased by 229% during Feedback. Arm-swing symmetry (R/L ROM ratio; Figure 3b) also differed across conditions (F(3,33)=4.13, p=0.014, $\eta_{p}^{2}=0.273$), although Bonferroni-adjusted pairwise comparisons were not significant (all p≥0.118). One-sample *t*-tests against perfect symmetry (R/L =1.0) indicated that symmetry did not differ from 1.0 in the Baseline, but was significantly greater than 1.0 in Exaggerated (p=0.010), Fast (p=0.013), and Feedback (p=0.046) conditions, with Feedback showing the smallest between-subject variability.

Gait outcomes showed condition-dependent changes in normalized walking speed and stride length (Figure 3c,d). Normalized walking speed differed significantly across conditions (F(3,33)=36.80, p<0.001, $\eta_{p}^{2}=0.770$). Post hoc comparisons indicated greater speed during Exaggerated, Fast, and Feedback relative to the Baseline (all p<0.001). Speed was greater during Fast (p<0.001) and Feedback (p=0.011) relative to Exaggerated, while Fast and Feedback did not differ (p=1.000). Normalized stride length also showed a significant main effect of the condition (F(3,33)=32.06, p<0.001, $\eta_{p}^{2}=0.745$). Post hoc comparisons indicated greater stride length during Exaggerated, Fast, and Feedback relative to the Baseline (all p<0.001), with Feedback exceeding Exaggerated (p=0.003), and no difference between Fast and Feedback (p=0.657). Cadence differed significantly across conditions (F(3,33)=38.61, p<0.001, $\eta_{p}^{2}=0.778$). Cadence was highest during Fast, exceeding the Baseline (p<0.001), Exaggerated (p<0.001), and Feedback (p=0.010). Feedback cadence also exceeded the Baseline (p=0.002) and did not differ from Exaggerated (p=0.300). Comprehensive descriptive statistics for all outcome measures, including additional variables (e.g., right- and left-arm ROM, cycle time, and absolute gait metrics), are provided in Appendix A Table A1; the absolute (non-normalized) gait metrics exhibited the same effects of walking conditions as the normalized metrics.

### 3.2. Feedback Condition: Direction and Magnitude Effects

**Figure 4:**
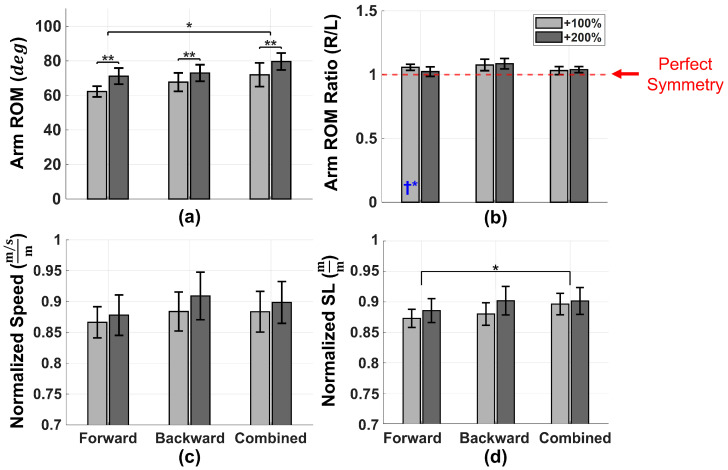
Outcomes across the six feedback conditions varying *Direction* (Forward, Backward, Combined) and *Magnitude* (+100%, +200%). Panels show (**a**) arm-swing ROM, (**b**) right-to-left arm ROM ratio (R/L), (**c**) normalized walking speed, and (**d**) normalized stride length (SL). Bars represent the mean ± SEM. Horizontal brackets indicate statistically significant Bonferroni-adjusted post hoc pairwise comparisons performed as follow-up tests to significant main effects from the two-way repeated-measures ANOVA (*Direction* × *Magnitude*) (* p<0.05, ** p<0.01). The dashed line in (**b**) indicates perfect symmetry (R/L = 1.0), and blue † symbols indicate significant deviation from 1.0 based on one-sample *t*-tests (* p<0.05).

The analyses above demonstrated that Feedback produced the greatest improvements in both arm-swing ROM and gait performance relative to Baseline, Exaggerated, and Fast conditions. To further examine whether different feedback conditions produced distinct effects, a factorial (two-way) repeated-measures ANOVA was conducted to test the independent contributions of feedback *Direction* (Forward vs. Backward vs. Combined), feedback *Magnitude* (+100% vs. +200% of Baseline targets), and their interaction across the six feedback conditions. The results are illustrated in Figure 4, and complete descriptive statistics for the six feedback conditions are provided in Appendix A Table A2.

Across feedback configurations, the Combined arm-swing ROM showed significant main effects of *Direction* and *Magnitude* (*Direction*: F(2,22)=7.17, p=0.004, $\eta_{p}^{2}=0.395$; *Magnitude*: F(1,11)=9.79, p=0.010, $\eta_{p}^{2}=0.471$), with no *Direction* × *Magnitude* interaction. Bonferroni-adjusted post hoc comparisons for the *Direction* main effect indicated a greater ROM for Combined versus Forward (p=0.033; Figure 4a), while Forward versus Backward (p=0.293) and Backward versus Combined (p=0.085) were not significant. Marginal means for the *Magnitude* main effect indicated a greater ROM under +200% than +100% targets when averaged across directions. Arm-swing symmetry (ROM ratio, R/L) was preserved across feedback configurations, with no main effects of *Direction*, *Magnitude*, or interaction (Figure 4b). One-sample *t*-tests against perfect symmetry (R/L =1.0) indicated that only Forward +100% deviated significantly from 1.0 (p=0.035), whereas all other feedback conditions did not differ from 1.0 (all p≥0.061).

Gait outcomes were primarily influenced by feedback *Direction*. Normalized stride length showed a significant main effect of *Direction* (F(2,22)=8.25, p=0.002, $\eta_{p}^{2}=0.429$), with post hoc comparisons indicating that Combined exceeded Forward (p=0.015; Figure 4d); other *Direction* comparisons were not significant. Normalized walking speed also showed a significant *Direction* effect (F(2,22)=3.80, p=0.038, $\eta_{p}^{2}=0.257$), but Bonferroni-adjusted pairwise comparisons did not reach significance (all p≥0.060; Figure 4c); neither outcome showed a significant main effect of *Magnitude* or a *Direction* × *Magnitude* interaction. Cadence did not differ significantly by *Direction* or *Magnitude*, with no *Direction* × *Magnitude* interaction.

Overall, the Combined arm-swing ROM was independently influenced by both feedback *Direction* and target *Magnitude*, whereas gait outcomes were primarily affected by feedback *Direction*, with no evidence of *Direction* × *Magnitude* interactions across outcomes.

## 4. Discussion

This study evaluated whether a fully portable, closed-loop vibrotactile feedback system could increase arm-swing amplitude during overground walking in community-dwelling older adults and whether these upper-limb changes would be accompanied by alterations in spatiotemporal gait. The primary hypothesis was that closed-loop vibrotactile feedback would produce larger increases in the arm-swing ROM than verbal instruction alone (Exaggerated and Fast) and that these increases would be accompanied by systematic changes in walking speed and stride length. The results supported this hypothesis. Feedback produced the largest increases in the arm-swing ROM (+229% relative to the Baseline), exceeding both Exaggerated and Fast conditions, and was accompanied by significant increases in walking speed and stride length relative to the Baseline. These findings indicate that amplitude-specific corrective vibrotactile feedback was more effective than instruction alone in driving arm-swing modulation and was associated with measurable whole-body gait adaptations. Importantly, these increases in arm-swing amplitude were achieved without substantial degradation of arm-swing symmetry at the group level. In contrast to the Exaggerated and Fast conditions, which showed significant right-dominant asymmetry, symmetry during Feedback remained closer to unity and exhibited lower between-subject variability. The secondary hypothesis was that feedback *Direction* and target *Magnitude* would independently influence arm-swing kinematics, such that Combined feedback would produce the greatest increases in the total arm ROM, and the +200% condition would elicit greater changes in kinematics and gait parameters than the +100% condition. This hypothesis was partially supported. Both *Direction* and *Magnitude* demonstrated independent main effects on the arm-swing ROM, and Combined feedback produced a greater ROM than Forward feedback, consistent with predictions. A higher target magnitude (+200%) also elicited greater increases in arm kinematics than +100%, supporting the predicted scaling effect. However, the predicted translation of magnitude-dependent scaling to spatiotemporal gait outcomes was not observed; gait parameters were more consistently influenced by feedback *Direction* than by target *Magnitude*, and no *Direction* × *Magnitude* interactions were detected. Together, these findings suggest that which phase of the arm swing is targeted (feedback *Direction*) has a greater impact on gait outcomes than increasing the amplitude target alone, whereas amplitude scaling mainly affects arm-swing kinematics.

### 4.1. Real-Time Vibrotactile Feedback and Arm-Swing Modulation

The magnitude of modulation achieved under Feedback warrants particular consideration. The arm-swing ROM increased by 229% relative to the Baseline, substantially exceeding both the increases observed in the no-feedback conditions and those reported in prior work using voluntary arm-swing exaggeration (∼150% increase) [29] or rhythm-based haptic cueing strategies (∼30% increase) in older adults [29]. Prior experimental studies indicate that even modest increases in arm-swing amplitude are sufficient to influence stride length and walking speed [20,23], and greater arm swing has been associated with reductions in mediolateral trunk motion and improved dynamic stability [30,31]. Although optimal amplitude targets for clinical benefit remain undefined, the magnitude of change observed here suggests that vibrotactile feedback can drive biomechanically meaningful upper-limb modulation in a population that commonly exhibits reduced spontaneous arm swing [15,19,44]. Importantly, arm-swing amplitude could be scaled in a predictable manner by adjusting feedback parameters. *Direction* and target *Magnitude* influenced the arm ROM independently, with no interaction between these factors. Increasing the amplitude target increased the ROM regardless of Direction, and Combined feedback produced a greater arm-swing ROM than Forward or Backward feedback, regardless of target Magnitude. From a practical perspective, this independence suggests that feedback parameters can be selected to achieve different levels of arm-swing modulation based on individual tolerance, Baseline arm-swing characteristics, and specific application context, without requiring complex parameter tuning. Moreover, although Exaggerated and Fast also increased arm-swing amplitude, both conditions showed significant right-dominant asymmetry. In contrast, the large amplitude increases achieved under Feedback did not produce systematic degradation of right–left symmetry at the group level and were associated with more consistent symmetry (smaller between-subject variability) across participants. Across the six feedback configurations, symmetry was preserved in all conditions except Forward +100%, which showed a small deviation from perfect symmetry. From a translational perspective, the ability to increase arm-swing amplitude without exacerbating asymmetry may be relevant for promoting consistent and potentially safer gait adaptations in older adults, as age-related arm-swing asymmetry has been associated with altered coordination and changes in gait control in aging and neurological populations [15,19,45].

### 4.2. Real-Time Vibrotactile Feedback and Spatiotemporal Gait Adaptations

Although the vibrotactile system was designed to target upper-limb kinematics, spatiotemporal gait parameters also changed across walking conditions. Increases in arm-swing amplitude under both instruction (Exaggerated, Fast) and Feedback were accompanied by corresponding increases in normalized walking speed and normalized stride length, with the largest gait changes occurring during Feedback. Under Feedback, walking speed increased by approximately 20%, and stride length increased by approximately 15% relative to the Baseline, indicating that upper-limb-focused real-time feedback was accompanied by measurable whole-body gait adaptations during overground walking. This pattern is consistent with evidence that arm swing is mechanically and neurally coupled to lower-limb gait mechanics [16,17,18,46], and that arm-swing kinematics are associated with stride length and walking speed [20,47]. Experimental studies further demonstrate that deliberately modifying arm swing can influence spatiotemporal outcomes during walking [20,23,29]. Although the present study was not designed to isolate the neuromotor mechanisms underlying these adaptations, the concurrent increases in the arm ROM, stride length, and walking speed suggest that amplifying arm swing can co-occur with measurable changes in gait performance during overground walking. These findings are also consistent with established arm–leg coupling during walking, supporting the interpretation that promoting arm swing may influence whole-body gait mechanics [20,48].

Walking speed can increase through two primary strategies: increasing cadence or increasing stride length [49]. In the present study, Exaggerated, Fast, and Feedback each increased speed relative to the Baseline, but did so using different combinations of cadence and stride length. Exaggerated walking showed a mixed strategy, with modest increases distributed across both cadence and stride length. Fast walking produced the clearest cadence-driven strategy, whereas Feedback achieved similar speed gains with comparatively larger stride length and lower cadence than Fast, suggesting a more stride-length-driven adaptation under closed-loop feedback. This distinction may matter for older adults. Although increasing stride length is often considered a more efficient locomotor strategy, older individuals with reduced stride length frequently rely on increased cadence as a compensatory mechanism to maintain walking speed, a pattern associated with frailty and mobility decline [50]. Increasing walking speed through longer strides rather than higher cadence may therefore be preferable for individuals who cannot safely or comfortably increase step frequency due to balance limitations, fatigue, or joint loading concerns [13,51]. The stride-length-driven speed increases observed under Feedback therefore suggest a potentially favorable adaptation strategy for enhancing walking performance in aging populations.

The feedback-configuration findings further suggest that how cues are distributed across the arm-swing cycle may influence whole-body gait adaptation. Gait outcomes varied more consistently with feedback *Direction* than with target *Magnitude*, indicating that providing cues in both swing directions (Combined) may support greater gait adaptation than simply increasing the amplitude target alone. One possible explanation is that bidirectional cueing promotes a more consistent arm-swing pattern across the gait cycle; however, this was not directly assessed in the present study. Future work should determine whether feedback-driven increases in stride length and walking speed are accompanied by changes in trunk motion, gait stability, variability, and perceived effort, and whether these adaptations persist after feedback removal.

### 4.3. Comparison with Other Wearable Vibrotactile Feedback Approaches

The present findings build on a growing body of work showing that wearable vibrotactile feedback can be used to systematically modify gait during overground walking. Lee et al. used vibrotactile bracelets to cue arm-swing changes across multiple protocols that varied the targeted arm(s), swing Direction, and movement phase [23]. In healthy young individuals, increasing arm-swing amplitude produced a clear increase in stride length, whereas walking speed showed smaller and less consistent changes, and was often not statistically significant [23]. Their results support the interpretation that arm-swing amplitude has a strong mechanical and coordination-based relationship with step length, while speed changes may depend more on the overall task constraints and how participants distribute the change across cadence and stride length. In contrast, the current study produced not only large increases in the arm ROM, but also consistent increases in stride length and speed under feedback. One possible reason is the difference in how feedback was delivered. In Lee et al., vibration mainly served as a confirmation cue (it turned on when participants reached the target arm angle) [23]. In contrast, our system provided a corrective cue (it turned on when arm swing fell below the target), which may have helped participants detect performance drops more quickly, adjust immediately, and maintain the desired arm-swing amplitude more consistently throughout the walking trial.

Our results also align with wearable vibrotactile approaches that target lower-limb kinematics to directly shape spatiotemporal gait outcomes. Sharafian et al. developed a real-time vibrotactile feedback system designed to increase peak thigh extension, a kinematic feature closely linked to stride length [13]. Their findings demonstrated that feedback-driven increases in thigh extension increased stride length and consequently walking speed in older adults, with changes comparable to those achieved through verbal instruction [13]. Together with the present arm-focused system, these studies suggest a common mechanism across wearable biofeedback approaches: reinforcing a specific biomechanical parameter in real time (whether thigh extension or arm ROM) can propagate to whole-body gait changes such as longer steps and higher speed.

Finally, relative to our prior rhythmic haptic cueing system that targeted temporal features of arm swing (arm cycle time) [12], the present amplitude-based feedback system produced substantially larger increases in the arm ROM while also improving spatiotemporal gait outcomes. In the rhythmic cueing paradigm, decreasing arm cycle time produced modest increases in the walking speed (+18.2%), stride length (+7.5%), and arm ROM (+30.2%) [12]. In contrast, the current amplitude-based feedback produced a markedly larger increase in the arm ROM (+229%), while walking speed and stride length increased by approximately 20% and 15%, respectively. Together, these results suggest that rhythmic cueing primarily facilitates speed-related gains by modulating the timing of arm movements, whereas amplitude-based feedback more directly increases arm-swing magnitude while still propagating to meaningful improvements in stride length and walking speed.

### 4.4. Limitations and Future Work

Several limitations should be considered. First, the sample size was modest (n=12), as this study represents an initial feasibility investigation, and participants were community-dwelling; larger cohorts are needed to assess generalizability and determine whether responsiveness depends on functional status or Baseline arm-swing characteristics. Second, this study examined within-session effects; longitudinal training studies are needed to evaluate retention (i.e., persistence under no-feedback conditions), transfer to daily walking, and cognitive load during feedback compared to no-feedback walking. Third, although gait speed and stride length improved under feedback, the present work did not directly quantify underlying mechanisms such as trunk rotation, arm–leg phase coordination, or neuromuscular responses; future work integrating trunk kinematics and electromyography could clarify how amplitude feedback modifies interlimb coordination. Finally, future studies should examine whether individualized, arm-specific targets (rather than using the better-performing arm to set targets) can further improve bilateral symmetry for participants with Baseline asymmetries, particularly in people who might have marked asymmetries due to a neurological lesion such as stroke.

### 4.5. Conclusions

This study demonstrated that real-time, vibrotactile feedback can robustly increase arm-swing amplitude during overground walking in community-dwelling older adults. Vibrotactile feedback produced the largest arm-swing modulation (229% increase from the Baseline) and was accompanied by increases in walking speed and stride length relative to the Baseline and verbal instruction conditions. Across feedback configurations, both *Direction* and *Magnitude* independently influenced arm-swing amplitude, while gait outcomes were primarily affected by *Direction*. Importantly, these gains were achieved without significant degradation of arm-swing symmetry at the group level. Together, these results support the feasibility of wearable IMU-driven vibrotactile biofeedback as an effective, portable approach to regulate or increase arm swing and influence whole-body gait mechanics in typical walking environments.

## Data Availability

The raw data supporting the conclusions of this article will be made available by the authors upon request, subject to privacy and ethical restrictions involving human participants.

## Abbreviations

The following abbreviations are used in this manuscript:

ABC	Activities-specific Balance Confidence
BLE	Bluetooth Low Energy
IMU	inertial measurement unit
R/L	right-to-left
ROM	range of motion
SEM	Standard Error of the Mean
SPPB	Short Physical Performance Battery
$\eta_{p}^{2}$	partial eta-squared

## Appendix A. Descriptive Statistics

This appendix provides comprehensive descriptive statistics (mean ± SD, n=12) for all outcome measures analyzed in this study. Table A1 summarizes results across the four primary walking conditions (Baseline, Exaggerated, Fast, and Feedback), where Feedback represents the participant-specific average across the six feedback configurations. Table A2 reports outcomes for each of the six feedback conditions, which varied systematically in feedback *Direction* (Forward, Backward, Combined) and feedback *Magnitude* (+100%, +200% of Baseline targets).

**Table A1:** Descriptive statistics across the four walking conditions (mean ± SD, n=12). ROM = range of motion; SL = stride length; SD = standard deviation.

Parameter	Baseline	Exaggerated	Fast	Feedback
*Arm-swing outcomes*				
Right-arm ROM (°)	22.34±8.74	51.35±15.22	39.22±8.34	72.46±15.16
Left-arm ROM (°)	20.79±7.22	43.50±16.77	31.68±12.84	69.41±16.41
Combined arm ROM (°)	21.57±7.06	47.43±15.66	35.45±9.56	70.93±15.60
ROM ratio (R/L)	1.101±0.361	1.231±0.258	1.362±0.423	1.052±0.080
*Gait parameters*				
Speed (m/s)	1.245±0.130	1.349±0.127	1.531±0.127	1.503±0.165
Normalized speed ($\frac{m/s}{m}$)	0.736±0.099	0.798±0.107	0.905±0.103	0.887±0.108
Stride length (m)	1.313±0.094	1.390±0.084	1.471±0.118	1.511±0.131
Normalized SL ($\frac{m}{m}$)	0.774±0.056	0.820±0.062	0.867±0.065	0.890±0.065
Cadence (steps/min)	113.7±7.7	116.4±7.6	125.0±7.4	119.2±6.8
Cycle time mean (s)	1.060±0.069	1.035±0.068	0.963±0.058	1.011±0.058
Cycle time SD (s)	0.019±0.004	0.018±0.004	0.015±0.003	0.017±0.004

**Table A2:** Descriptive statistics across the six feedback conditions (mean ± SD, n=12). ROM = range of motion; SL = stride length; SD = standard deviation.

Parameter	Forward	Forward	Backward	Backward	Combined	Combined
	+100%	+200%	+100%	+200%	+100%	+200%
*Arm-swing outcomes*						
Right ROM (°)	63.77±10.43	71.71±16.63	69.62±18.62	75.49±16.98	73.16±23.87	81.00±17.33
Left ROM (°)	60.69±11.40	70.61±16.82	65.71±19.30	70.45±17.50	70.72±23.83	78.28±17.37
Combined ROM (°)	62.23±10.69	71.16±16.20	67.67±18.55	72.97±16.62	71.94±23.73	79.64±17.04
ROM ratio (R/L)	1.058±0.083	1.023±0.130	1.076±0.155	1.085±0.142	1.032±0.109	1.039±0.084
*Gait parameters*						
Speed (m/s)	1.469±0.127	1.489±0.175	1.498±0.161	1.542±0.214	1.498±0.177	1.524±0.186
Normalized speed ($\frac{m/s}{m}$)	0.866±0.087	0.878±0.114	0.884±0.109	0.909±0.134	0.883±0.114	0.899±0.117
Stride length (m)	1.483±0.106	1.504±0.131	1.494±0.119	1.532±0.159	1.524±0.135	1.532±0.153
Normalized SL ($\frac{m}{m}$)	0.873±0.052	0.886±0.068	0.880±0.064	0.902±0.081	0.896±0.061	0.901±0.076
Cadence (steps/min)	118.8±6.5	118.5±6.8	120.1±7.7	120.4±7.8	117.8±8.2	119.2±6.9
Cycle time mean (s)	1.013±0.057	1.016±0.058	1.003±0.066	1.000±0.065	1.023±0.071	1.010±0.059
Cycle time SD (s)	0.014±0.003	0.017±0.004	0.016±0.004	0.018±0.005	0.018±0.006	0.018±0.005
